# Capture of Pb^2+^ and Cu^2+^ Metal Cations by *Neisseria meningitidis*-type Capsular Polysaccharides

**DOI:** 10.3390/biom8020023

**Published:** 2018-05-05

**Authors:** Sujan Ghimire, Pumtiwitt C. McCarthy

**Affiliations:** Department of Chemistry, Morgan State University, Baltimore, MD 21251, USA; Sujan.Ghimire@morgan.edu

**Keywords:** capsular polysaccharide, *Neisseria meningitidis*, bioremediation, heavy metals

## Abstract

Heavy metal pollution of water is a significant environmental and public health concern. Current biological strategies for heavy metal removal from water are performed using microbial biopolymers, including polysaccharides, that are already fully formed. This creates limitations in adapting polysaccharides to increase binding affinity for specific metals. We propose that altering the specificity of polysaccharide-producing enzymes could be beneficial to improving metal capture by modified polysaccharides. We assess binding of Cu^2+^ and Pb^2+^ metal cations to *Neisseria meningitidis*-type polysaccharides. All concentrations of metal cations tested were able to completely bind to colominic acid. This polymer is equivalent to the capsular polysaccharide of *N. meningitidis* serogroup B comprised of a homopolymer of negatively charged sialic acid. There was slightly less binding observed with *N. meningitidis* serogroup W, which contains repeating units of the neutral sugar galactose and sialic acid. Our work represents the first assessment of the metal-binding properties of these capsular polysaccharides. Future work will seek to optimize metal-binding with *Neisseria meningitidis* serogroup W polysaccharide.

## 1. Introduction

Water pollution by heavy metals is an environmental and public health risk [1]. Polluted water, usually caused by industrial waste byproducts, can negatively impact natural habitats of marine animals leading to disruption of ecosystems on which humans rely [2]. In terms of public health, seafood or plants obtained from polluted sources for consumption and metal-polluted water is detrimental to human health. Metal poisoning can severely damage nearly all cellular components [3,4,5,6,7,8,9]. Consequently, effective heavy metal removal from affected areas is extremely important. There has been an increase in the development of new heavy-metal capture methods that are eco-friendly. Bioremediation is a process that uses living organisms (mostly microorganisms and plants) rather than harsh chemicals to remove and/or detoxify waste products and pollutants [10,11]. Microorganisms perform bioremediation either via biosorption or bioaccumulation. Biosorption is the removal of heavy metals by passive binding to non-living biomass in an aqueous solution. Alternatively, bioaccumulation is an active process that requires the metabolic activity of a living organism in the removal of metals. Many examples of both methods exist in the literature.

Microbial extracellular polymeric substances can play key roles in biosorption. Bacteria, fungi and some algae are known to produce exopolysaccharides [12]. Exopolysaccharides contain mostly polysaccharides, but can also contain nucleic acids, protein and phospholipids. Extracellular polymeric substances are one component of exopolysaccharides [13]. These polysaccharides can be found released into the environment or attached to the microorganism cell surface (in the case of capsular polysaccharides). Microbial extracellular polymeric substances play physiological roles in cell adhesion, biofilm formation and protection from host defense mechanisms [14,15]. These biopolymers are equipped with ionizable functional groups that are known sites for interactions with heavy metal cations. These include groups such as carboxylic (–COOH), phosphoryl (–PO_4_), amino (–NH_3_) and hydroxyl (–OH) groups [16]. Many studies have investigated metal binding properties from diverse microorganisms such as *Marinobacter* species, *Azotobacter beijreinckii*, *Bacillus subtilis*, and others [17,18,19].

**Figure 1:**
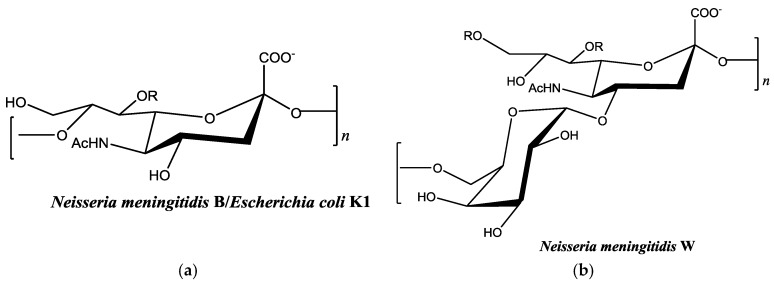
Chemical structures of monosaccharide units of bacterial polysaccharides used in this study [28,29]. (**a**) Both capsular polysaccharides contain α-2,8 linked sialic acid; and (**b**) the α-1,4 linked galactose-sialic acid heteropolymer. R in each figure represents potential sites of acetylation in vivo.

There are relatively few published studies, however, that seek to optimize the metal-binding properties of an organism’s capsular polysaccharide using the enzymatic machinery responsible for polysaccharide synthesis [20]. Most investigation of metal-binding properties is performed on biopolymers that are already fully formed. This creates limitations in adapting polysaccharides to increase binding affinity for specific metals. Genetic engineering and recombinant DNA technology make it possible to design and optimize new biopolymers for this purpose. Our long-term goal is to optimize the binding properties of the polysaccharide from *Neisseria meningitidis* serogroup W for this purpose (Figure 1a). This bacterium is one of six types of disease-causing serogroups of *N. meningitidis*. The capsular polysaccharide-producing enzyme from serogroup W and other serogroups are all available through recombinant methods [21,22,23,24,25,26]. This availability makes it possible to optimize these enzymes for polysaccharide synthesis in vitro. Most studies with serogroup W capsular polysaccharides have focused on vaccine development [27]. However, based on the polysaccharide structure, it may bind strongly to heavy metal cations. Efforts in this research laboratory are focused on optimizing activity of this enzyme and investigating its substrate specificity. Potentially, modified polysaccharides with new metal-binding properties could be enzymatically synthesized using non-natural carbohydrate substrates. In this work, we describe for the first time the metal-binding properties of *N. meningitidis*-type polysaccharides (Figure 1). We evaluate binding of Cu^2+^ and Pb^2+^ with colominic acid (equivalent to the α-2,8 linked sialic acid homopolymer of *N. meningitidis* serogroup B) and *N. meningitidis* serogroup W polysaccharide (a heteropolymer of repeating units of α-1,4 linked galactose-sialic acid).

## 2. Results and Discussion

### 2.1. Free Metal Cations Are Sequestered by a Polymeric Resin

**Figure 2:**
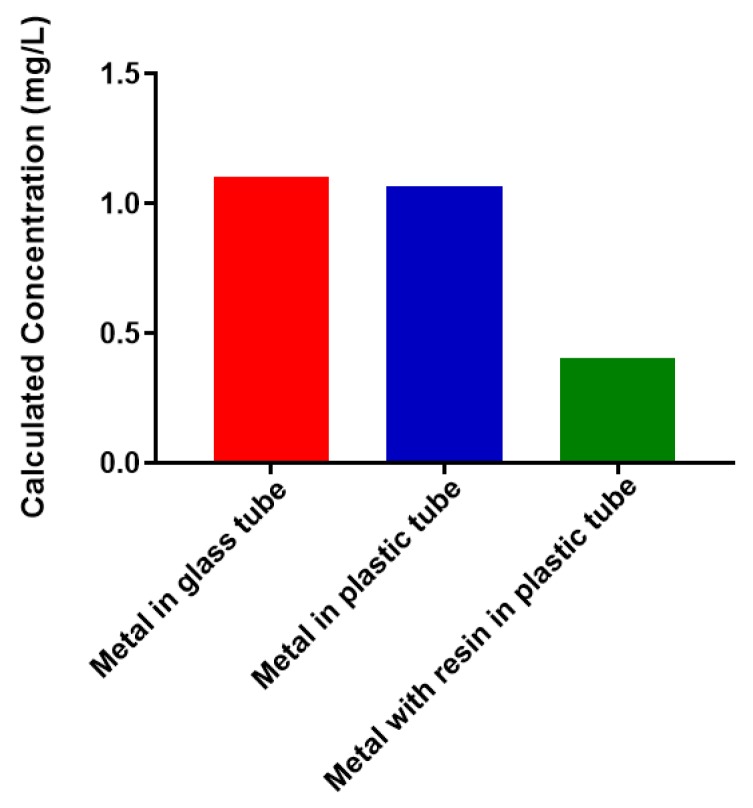
Free Pb^2+^ concentration under various conditions. Free metal was detected by atomic absorption spectroscopy.

Bacterial polysaccharides have been shown to have strong metal-binding properties. One original goal of this work was to use recombinant *N. meningitidis* serogroup W capsule polymerase to create a form of the polysaccharide that could be conjugated to a polymeric support using click chemistry [30]. This could provide a potential new avenue for removal of heavy metals from aqueous solutions. We performed a selection of experiments to determine feasibility. We wanted to determine the best reaction vessel to conduct experiments in and whether the polymeric support had any interactions with free, unconjugated Pb^2+^ (Figure 2). The amount of free metal present (determined by atomic absorption spectroscopy, AAS) was the same before incubation (1 mg/L) and after a 1 h incubation for both glass and plastic. This indicates that there was no interaction with the container that removes the metal out of solution. However, when the same amount of free metal is incubated in plastic in the presence of a 6% alkyne-agarose support, there is a dramatic decrease in the amount of free metal present. This suggests that a large percentage of the free metal enters the resin and is inaccessible to detection by AAS. Therefore, use of the selected polymeric support for attachment of polysaccharides would not prove fruitful. Future work will investigate inert materials for conjugation to polysaccharides. We are moving forward towards assessing the metal-binding properties of the selected bacterial polysaccharides because no assessment exists in the literature.

### 2.2. Colominic Acid Binds High Concentrations of Pb^2+^ and Cu^2+^

*Neisseria meningitidis* serogroup B is a homopolymer of α-2,8-linked *N*-acetylneuraminic acid (Figure 1a). This is the same structure found in the capsule of *Escherichia coli* K1 [31]. Antibodies against capsular polysaccharide of *E. coli* K1 are cross-reactive with *N. meningitidis* serogroup B capsular polysaccharide [32]. By investigating metal-binding to colominic acid we are indirectly measuring binding to *Neisseria meningitidis* serogroup B. Colominic acid was used because it is commercially available. We investigated the binding of 1 mg/mL colominic acid to 5–50 mg/L of Pb^2+^ and Cu^2+^ cations, respectively.

**Figure 3:**
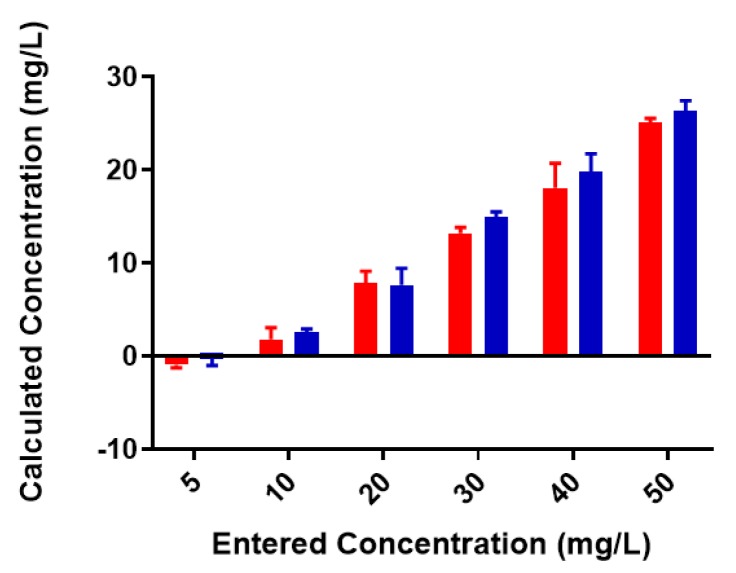
Pb^2+^ concentrations in unfiltered control and reaction samples; red = control (no colominic acid), blue = reaction (with colominic acid). Entered concentration refers to the metal concentration of the prepared working solutions.

**Figure 4:**
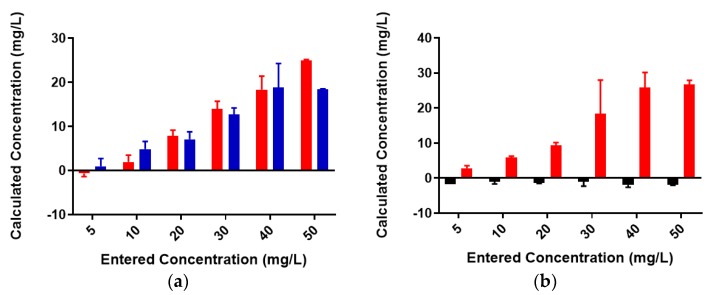
Lead-binding to colominic acid. (**a**) Pb^2+^ concentrations in filtrate (red) and retentate (blue) of control samples and (**b**) reaction samples; filtrate is in black and retentate is in red. Entered concentration refers to the metal concentration of the prepared working solutions.

A standard curve for each metal was created using AAS standards. The standard curve for lead (Figure S1) was found to be linear through the entire range of concentrations (2–80 mg/L). Metal-binding was assessed after 2 h incubation to obtain equilibrium for control (no colominic acid) and reaction samples (with colominic acid) (see Materials and Methods). This differs slightly from Loaëc et al. [33], in which a 3 h incubation period was used to reach equilibrium in metal-binding experiments with exopolysaccharides. After this time, 50% of these reactions (3 mL of 6 mL total) were passed through a 3 kDa cutoff filtration device. Polysaccharides and anything complexed to the polysaccharide will remain in the retentate and any free metal will pass through the filter. The free metal concentration was determined for both unfiltered and filtered control and reaction samples. In the testing of Pb^2+^ metal binding, the same initial concentration of metal was found to be present in both unfiltered control and reaction samples (Figure 3). These unfiltered samples tested were the remaining 50% (3 mL) that did not undergo filtration. The observed results indicate that the initial metal concentration is the same for both conditions. For filtered control samples, equal concentrations of metals were found to be present in both the filtrate and supernatant, indicating that unbound metals were freely able to pass through the filter (Figure 4a). In the case of reaction samples after filtration (Figure 4b) no metal was found to be present in filtrate, indicating formation of a polysaccharide–metal complex. This complex is not able to pass through the filter. All metal was polysaccharide-bound because the only metal present was found in the supernatant. The metal content of colominic acid alone was also tested, and no metal was present (results not shown), which suggests that any metal found in the supernatant was there because it was bound to polysaccharide.

**Figure 5:**
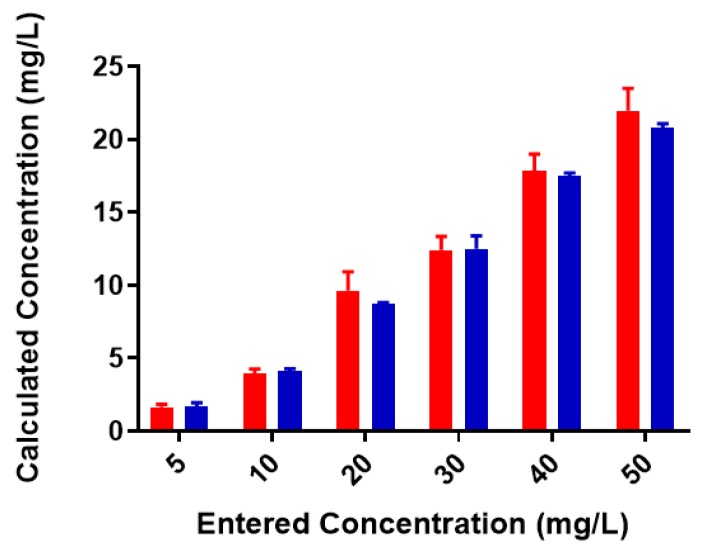
Cu^2+^ concentrations in unfiltered control and reaction samples; red = control (no colominic acid), blue = reaction (with colominic acid). Entered concentration refers to the metal concentration of the prepared working solutions.

Most of the same trends were observed for binding studies with Cu^2+^. However, one major difference can be found in one of the standard curves. This curve (Figure S2), used to determine the concentration in the case of Cu^2+^ ion, was not completely linear as in the case of Pb^2+^ ion (Figure S1). Copper was only linear from 2–20 mg/L, leading to a poorer linear fit and lower-than-expected values at experimental metal concentrations above this. While the instrument calculates metal concentration using the linear fit, a polynomial equation fits better to this data (Figure S3). Despite this, we see trends similar to what was observed in lead-binding studies. As seen previously, similar initial concentrations of metal were present in both unfiltered control and reaction samples (Figure 5).

**Figure 6:**
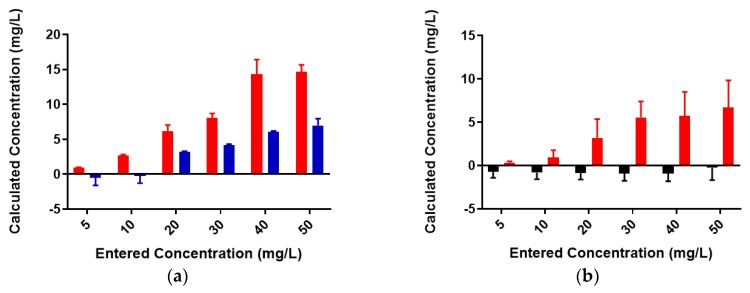
Copper-binding to colominic acid. Cu^2+^ concentrations in filtrate (red) and retentate (blue) of (**a**) control samples and (**b**) reaction samples; filtrate is in black and retentate is in red. Entered concentration refers to the metal concentration of the prepared working solutions.

The observed results for copper were like those seen for lead; however, there was less free metal in the supernatant compared to filtrate in the filtered control samples (Figure 6a). As before, no metal was found to be present in filtrate indicating formation of a complex (Figure 6b). All metal was present only in the supernatant. The observed overall copper concentrations here are lower than expected. This is likely due to the poor linear fit of one standard curve (Figure S2). However, it is evident that colominic acid completely binds both Pb^2+^ and Cu^2+^ at all concentrations tested in these studies, including the lowest (5 mg/L).

### 2.3. Neisseria meningitidis Serogroup W Capsular Polysaccharide Binds a High Concentration of Pb^2+^

**Figure 7:**
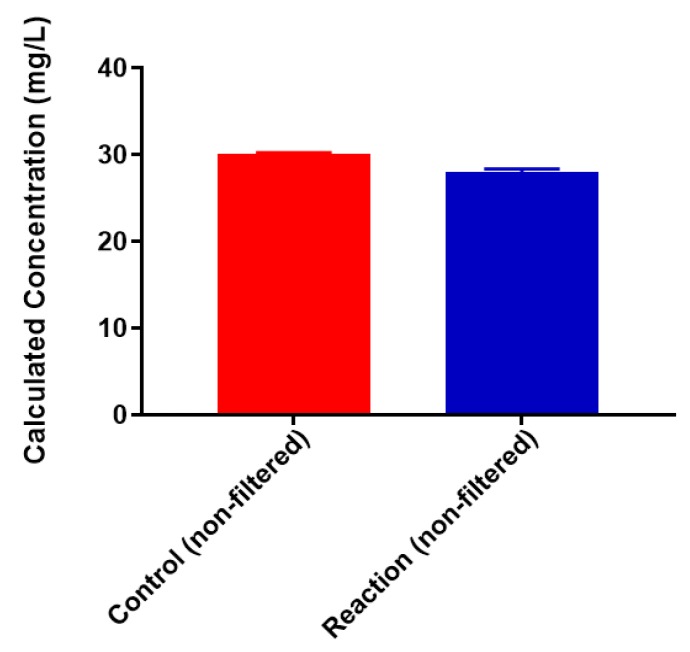
Pb^2+^ concentrations in unfiltered control and reaction samples; red = control (no serogroup W polysaccharide), blue = reaction (with serogroup W polysaccharide).

**Figure 8:**
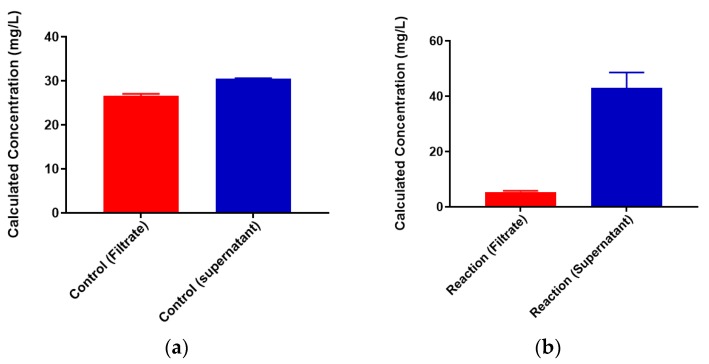
Lead (50 mg/L)-binding to serogroup W capsular polysaccharide. (**a**) Pb^2+^ concentrations in filtrate (red) and retentate (blue) of control samples and (**b**) reaction samples; filtrate is in red and retentate is in blue. Entered concentration refers to the metal concentration of the prepared working solutions.

Colominic acid contains only a repeating unit of negatively charged sialic acid, whereas the polysaccharide of serogroup W contains repeating unit of both neutral sugar galactose and negatively charged sialic acid. We wanted to assess the metal-binding of the serogroup W polysaccharide and used the highest concentration of lead metal for initial studies. The same trends that were observed with lead-binding to colominic acid were observed with this polysaccharide. The standard curve was linear, as expected (Figure S4). As before, the same initial concentration of metal was found to be present in both the unfiltered control and reaction samples (Figure 7). In the filtered control samples, equal concentrations of Pb^2+^ ion were found to be present in both the filtrate and the supernatant (Figure 8a). In reaction samples, unlike colominic acid, some Pb^2+^ cations (average from both trials = 5.4 mg/L) were found to be present in the filtrate, and an average of 43.0 mg/mL was found in the supernatant (Figure 8b). This might be due to the difference in composition of the two polysaccharides. There are more negatively charged functional groups in colominic acid to bind metal. We observed that there was no unbound metal to pass through the filter with that polysaccharide. The serogroup W polysaccharide has fewer negatively charged functional groups to bind the cations, which may explain why some unbound metal appeared in the filtrate (Figure 1).

## 3. Materials and Methods

### 3.1. Metal Capture by Alkyne-Modified Solid Support

A 6% agarose resin containing alkyne groups (Click Chemistry Tools, Scottsdale, AZ, USA) was tested for any interaction with Pb^2+^ cations. A 1 mg/L solution of lead(II) nitrate (5 mL total) (Sigma Aldrich, St. Louis, MO, USA) was taken in a 12 mL glass screwtop vial (VWR, Radnor, PA, USA) and was not incubated with resin. The same concentration of lead (II) nitrate was taken in a 15 mL screw-capped plastic tube (Genesee Scientific, San Diego, CA, USA) and incubated for 1 h by turning end over end (room temperature). Additionally, 1 mg/L (5 mL) of lead(II) nitrate was taken in a 15 mL plastic tube and mixed with 1 mL of resin and incubated for 1 h by turning end over end (room temperature). This sample was centrifuged, and the supernatant was used for metal determination. The concentration of Pb^2+^ ion in all three samples was analyzed by atomic absorption spectroscopy (Perkin Elmer Instruments Analyst 8000, Perkin Elmer, Waltham, MA, USA).

### 3.2. Binding of Heavy Metal Cations to Polysaccharide

The method performed was similar to that in a previously published procedure [33]. Atomic absorption spectrometry lead standard (1000 mg/L) in 2% HNO_3_ (*w*/*w*) was purchased from Sigma Aldrich (St. Louis, MO, USA). The working standard solutions (2 mg/L, 5 mg/L, 10 mg/L, 20 mg/L, 30 mg/L, 40 mg/L, and 80 mg/L) were made by appropriate dilutions from standard in 2% HNO_3_ (*w*/*w*). For the determination of metal binding to colominic acid (Sigma Aldrich), 1 mg/mL of colominic acid was made by dissolving 0.01 g of colominic acid in 10 mL of ultrapure, distilled water. A stock concentration of lead (250 mg/L) was made by dissolving 0.0025 g of lead(II) nitrate (Sigma Aldrich) in 10 mL of ultrapure, distilled water. Six different working concentrations (5 mL each) of lead (5 mg/L, 10 mg/L, 20 mg/L, 30 mg/L, 40 mg/L and 50 mg/L) were made by appropriate dilutions of the stock solution. Each sample was incubated with either 1 mL of ultrapure filtered water (controls) or with 1 mL of colominic acid (reactions). The experiment was performed in duplicate. Both controls and reactions were shaken at 200 rpm for 2 h at room temperature. After 2 h, a total of 3 mL of sample was passed through an Ultracel-3 membrane, 3 kDa cutoff (Sigma Aldrich) via centrifugation for 20 min at 6000 rpm. After centrifugation, the lead concentration in the filtrate, supernatant and unfiltered samples was analyzed using an atomic absorption spectrometer (Perkin Elmer Instruments Analyst 8000, Perkin Elmer, Waltham, MA, USA). A standard curve was prepared for each trial, and this was used by the instrument to quantitate the metal concentrations from all samples. Standard curves appear in Figures S1–S4. The same experimental methods were used for copper(II) nitrate (Sigma Aldrich). The same method used for colominic acid, described above, was used for *Neisseria meningitidis* serogroup W polysaccharide (a generous gift from Dr. Willie Vann, FDA/CBER) and lead. The only modification was that only the highest concentration of lead (50 mg/L) was assessed for binding.

## 4. Conclusions

The overall goal of this study was to determine the metal-binding capacity of capsular polysaccharides of two serogroups of *Neisseria meningitidis*, serogroup B (tested indirectly by colominic acid) and serogroup W with Pb^2+^ and Cu^2+^ cations. Colominic acid completely bound all metal concentrations. However, serogroup W polysaccharide was less efficient, as some unbound Pb^2+^ was detected. This polysaccharide has fewer negatively charged functional groups, which may account for this result. Future work will confirm which functional groups are involved in metal-binding by comparison of Fourier transform infrared spectroscopy (FTIR) spectrums of polysaccharide in the absence and presence of metal. In addition, more metal cations will be assessed for binding. *Neisseria meningitidis* serogroup B oligosaccharides have been suggested to adopt helical structures using computational modeling and nuclear magnetic resonance (NMR) spectroscopy [34]. Similar modeling in the presence of metal cations will be performed to assess whether there might be structural elements present that could affect binding.

## Supplementary Materials

The following are available online at http://www.mdpi.com/2218-273X/8/2/23/s1, Figure S1: Calibration curves for Pb^2+^ binding to colominic acid, Figure S2: Calibration curves for Cu^2+^ binding to colominic acid, Figure S3: Second order polynomial fit to Trial 2 standard curve for Cu^2+^ binding studies, Figure S4: Calibration curves for Pb^2+^ binding to serogroup W polysaccharide.

## References

[1] Tchounwou P.B., Yedjou C.G., Patlolla A.K., Sutton D.J. (2012). Heavy metal toxicity and the environment. Molecular, Clinical and Environmental Toxicology.

[2] Blewett T.A., Leonard E.M. (2017). Mechanisms of nickel toxicity to fish and invertebrates in marine and estuarine waters. Environ. Pollut..

[3] Tamas M.J., Fauvet B., Christen P., Goloubinoff P. (2018). Misfolding and aggregation of nascent proteins: A novel mode of toxic cadmium action in vivo. Curr. Genet..

[4] Zefferino R., Piccoli C., Ricciardi N., Scrima R., Capitanio N. (2017). Possible mechanisms of mercury toxicity and cancer promotion: Involvement of gap junction intercellular communications and inflammatory cytokines. Oxid. Med. Cell. Longev..

[5] Wessling-Resnick M. (2017). Excess iron: Considerations related to development and early growth. Am. J. Clin. Nutr..

[6] Song X., Fiati Kenston S.S., Kong L., Zhao J. (2017). Molecular mechanisms of nickel induced neurotoxicity and chemoprevention. Toxicology.

[7] Rinaldi M., Micali A., Marini H., Adamo E.B., Puzzolo D., Pisani A., Trichilo V., Altavilla D., Squadrito F., Minutoli L. (2017). Cadmium, organ toxicity and therapeutic approaches: A review on brain, kidney and testis damage. Curr. Med. Chem..

[8] Cicero C.E., Mostile G., Vasta R., Rapisarda V., Signorelli S.S., Ferrante M., Zappia M., Nicoletti A. (2017). Metals and neurodegenerative diseases. A systematic review. Environ. Res..

[9] Bulcke F., Dringen R., Scheiber I.F. (2017). Neurotoxicity of copper. Adv. Neurobiol. Y.

[10] Ojuederie O., Babalola O. (2017). Microbial and plant-assisted bioremediation of heavy metal polluted environments: A review. Int. J. Environ. Res. Public Health.

[11] Ayangbenro A.S., Babalola O.O. (2017). A new strategy for heavy metal polluted environments: A review of microbial biosorbents. Int. J. Environ. Res. Public Health.

[12] Nwodo U.U., Green E., Okoh A.I. (2012). Bacterial exopolysaccharides: Functionality and prospects. Int. J. Mol. Sci..

[13] Flemming H.C., Neu T.R., Wozniak D.J. (2007). The EPS matrix: The “house of biofilm cells”. J. Bacteriol..

[14] Corbett D., Roberts I.S. (2009). The role of microbial polysaccharides in host-pathogen interaction. F1000 Biol. Rep..

[15] Limoli D.H., Jones C.J., Wozniak D.J. (2015). Bacterial extracellular polysaccharides in biofilm formation and function. Microbiol. Spectr..

[16] Gupta P., Diwan B. (2017). Bacterial exopolysaccharide mediated heavy metal removal: A review on biosynthesis, mechanism and remediation strategies. Biotechnol. Rep..

[17] Poli A., Anzelmo G., Nicolaus B. (2010). Bacterial exopolysaccharides from extreme marine habitats: Production, characterization and biological activities. Mar. Drugs.

[18] Chug R., Gour V.S., Mathur S., Kothari S.L. (2016). Optimization of extracellular polymeric substances production using *Azotobacter beijreinckii* and *Bacillus subtilis* and its application in chromium (vi) removal. Bioresour. Technol..

[19] Bhaskar P.V., Bhosle N.B. (2006). Bacterial extracellular polymeric substance (eps): A carrier of heavy metals in the marine food-chain. Environ. Int..

[20] Schmid J. (2018). Recent insights in microbial exopolysaccharide biosynthesis and engineering strategies. Curr. Opin. Biotechnol..

[21] Fiebig T., Berti F., Freiberger F., Pinto V., Claus H., Romano M.R., Proietti D., Brogioni B., Stummeyer K., Berger M. (2014). Functional expression of the capsule polymerase of *Neisseria meningitidis* serogroup X: A new perspective for vaccine development. Glycobiology.

[22] Fiebig T., Freiberger F., Pinto V., Romano M.R., Black A., Litschko C., Bethe A., Yashunsky D., Adamo R., Nikolaev A. (2014). Molecular cloning and functional characterization of components of the capsule biosynthesis complex of *Neisseria meningitidis* serogroup A: Toward in vitro vaccine production. J. Biol. Chem..

[23] Ming S.A., Cottman-Thomas E., Black N.C., Chen Y., Veeramachineni V., Peterson D.C., Chen X., Tedaldi L.M., Wagner G.K., Cai C. (2018). Interaction of *Neisseria meningitidis* group X *N*-acetylglucosamine-1-phosphotransferase with its donor substrate. Glycobiology.

[24] Muindi K.M., McCarthy P.C., Wang T., Vionnet J., Battistel M., Jankowska E., Vann W.F. (2014). Characterization of the meningococcal serogroup X capsule *N*-acetylglucosamine-1-phosphotransferase. Glycobiology.

[25] Peterson D.C., Arakere G., Vionnet J., McCarthy P.C., Vann W.F. (2011). Characterization and acceptor preference of a soluble meningococcal group C polysialyltransferase. J. Bacteriol..

[26] Romanow A., Haselhorst T., Stummeyer K., Claus H., Bethe A., Muhlenhoff M., Vogel U., von Itzstein M., Gerardy-Schahn R. (2013). Biochemical and biophysical characterization of the sialyl-/hexosyltransferase synthesizing the meningococcal serogroup W135 heteropolysaccharide capsule. J. Biol. Chem..

[27] McCarthy P.C., Sharyan A., Sheikhi Moghaddam L. (2018). Meningococcal vaccines: Current status and emerging strategies. Vaccines.

[28] Bhattacharjee A.K., Jennings H.J., Kenny C.P., Martin A., Smith I.C. (1975). Structural determination of the sialic acid polysaccharide antigens of *Neisseria meningitidis* serogroups B and C with carbon 13 nuclear magnetic resonance. J. Biol. Chem..

[29] Bhattacharjee A.K., Jennings H.J., Kenny C.P., Martin A., Smith I.C. (1976). Structural determination of the polysaccharide antigens of *Neisseria meningitidis* serogroups Y, W-135, and BO1. Can. J. Biochem..

[30] McCarthy P.C., Saksena R., Peterson D.C., Lee C.H., An Y., Cipollo J.F., Vann W.F. (2013). Chemoenzymatic synthesis of immunogenic meningococcal group C polysialic acid-tetanus Hc fragment glycoconjugates. Glycoconj. J..

[31] Andreishcheva E.N., Vann W.F. (2006). Gene products required for de novo synthesis of polysialic acid in *Escherichia coli* K1. J. Bacteriol..

[32] Park I.H., Lin J., Choi J.E., Shin J.S. (2014). Characterization of escherichia coli k1 colominic acid-specific murine antibodies that are cross-protective against *Neisseria meningitidis* groups B, C, and Y. Mol. Immunol..

[33] Loaëc M., Olier R., Guezennec J. (1997). Uptake of lead, cadmium and zinc by a novel bacterial exopolysaccharide. Water Res..

[34] Battistel M.D., Shangold M., Trinh L., Shiloach J., Freedberg D.I. (2012). Evidence for helical structure in a tetramer of α2–8 sialic acid: Unveiling a structural antigen. J. Am. Chem. Soc..

