# Supplementary material for: Capture of Pb2+ and Cu2+ Metal Cations by Neisseria meningitidis-type Capsular Polysaccharides
Source: Biomolecules. 2018 May 5;8(2):23. doi: 10.3390/biom8020023 (PMC6023028; doi:10.3390/biom8020023)
Supplement: Supplementary file 1 [file biomolecules-08-00023-s001.pdf]

A)

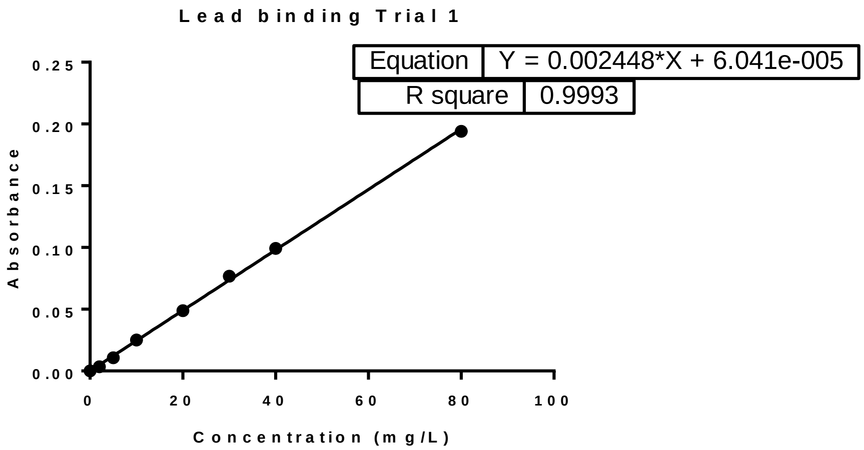

B)

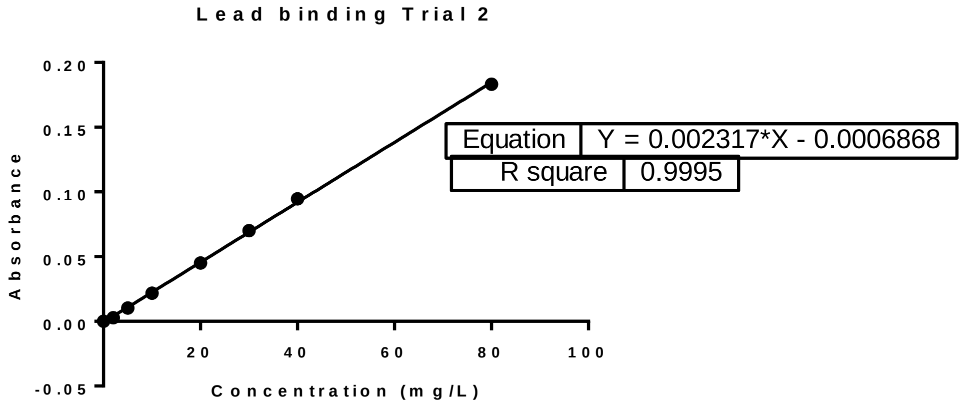

Fig. S1 Calibration curves for Pb2+ binding to colominic acid in A) Trial 1 and B) Trial 2. These linear regression curves to determine calculated concentrations during metal binding experiments

A)

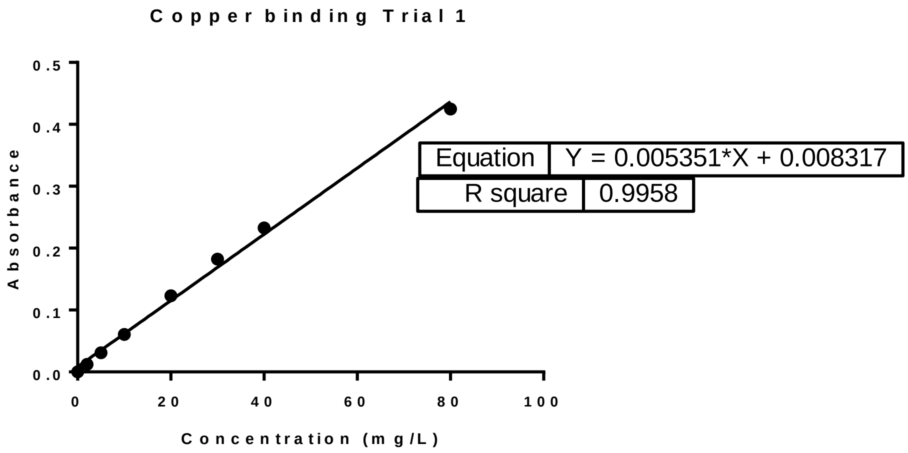

B)

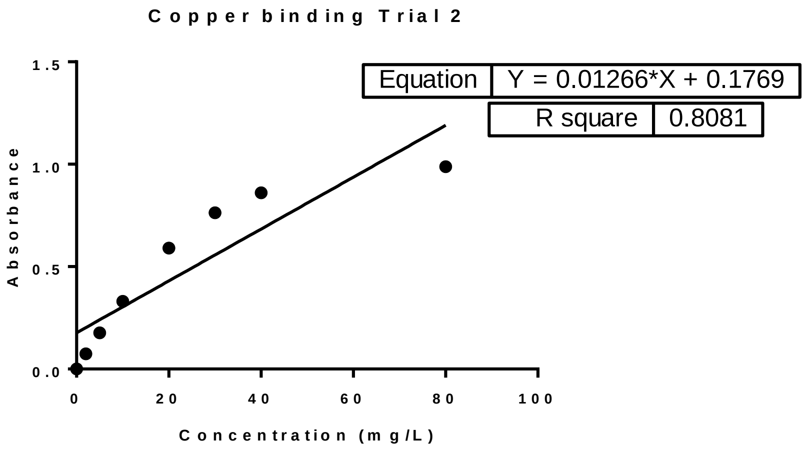

Fig. S2 Calibration curves for Cu^{2+} binding to colominic acid in A) Trial 1 and B) Trial 2. These linear regression curves to determine calculated concentrations during metal binding experiments

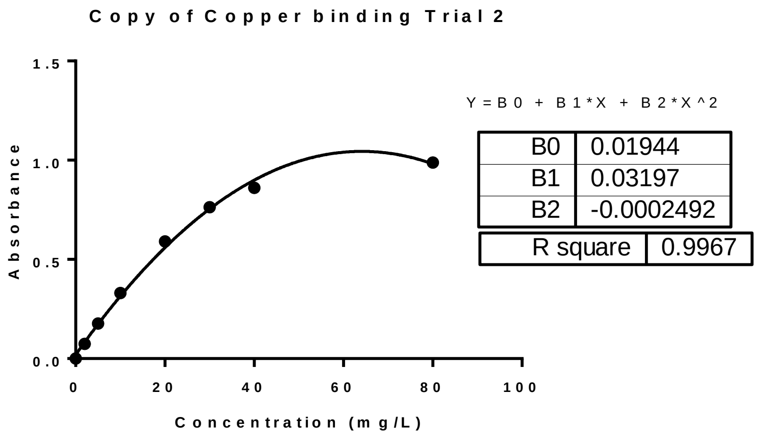

Fig. S3 Second order polynomial fit to Trial 2 standard curve data.

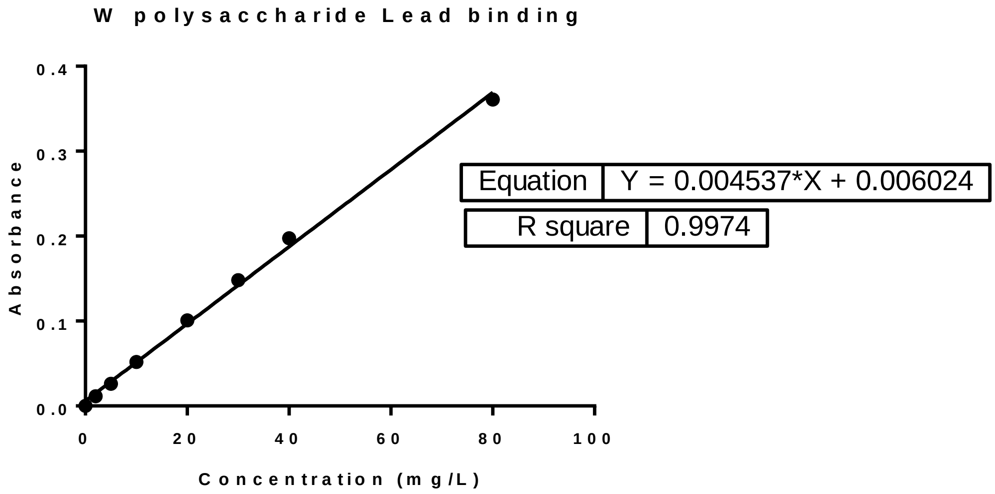

Fig. S4 Calibration curves for Pb^{2+} binding to serogroup W polysaccharide. The linear regression curves to determine calculated concentrations during metal binding experiments
